# Exploring the Potential Mechanisms of Danshen for the Treatment of Ulcerative Colitis based on Serum Pharmacochemistry, Gene Expression Profiling, and Network Pharmacology: Regulation of Cell Apoptosis and Inflammatory Response

**DOI:** 10.2174/0115734099318174240926103444

**Published:** 2024-10-10

**Authors:** Run-Xiang Zhai, Meng-Yu Wang, Hai-Tao Du, Chun-Xiao Yan, Zi-Wei Li, Kuo Xu, Hui Li, Xian-Jun Fu, Xia Ren

**Affiliations:** 1 Marine Traditional Chinese Medicine Research Center, Qingdao Academy of Traditional Chinese Medicine, Shandong University of Traditional Chinese Medicine, Qingdao, 266114, China;; 2 Qingdao Key Technology Innovation Center of Marine Traditional Chinese Medicine Deep Development and Industrialization, Qingdao, 266114, China;; 3 State Key Laboratory of Quality Research in Chinese Medicine, Macau University of Science and Technology, Taipa, Macau, China;; 4 College of Pharmacy, Shandong University of Traditional Chinese Medicine, Jinan, 250355, China

**Keywords:** Danshen, ulcerative colitis, serum pharmacochemistry, gene expression profiling, network pharmacology, cell apoptosis

## Abstract

**Background:**

As a traditional Chinese medicine, Danshen shows potential efficacy for treating ulcerative colitis (UC). However, the bioactive components and mode of action were unclear.

**Aim:**

This paper uses a combination of network pharmacology, serum medicinal chemistry, and gene expression profiling to clarify its possible molecular mechanism of action and material basis.

**Methods:**

Ultra-high performance liquid chromatography-mass spectrometry (UPLC-MS) was utilized to analyze the herbal components and metabolites from the serum of Danshen-treated mice. Gene expression profiles were applied to construct a database of Danshen action targets. Then, active ingredient-target-biological functional module networks were constructed to analyze the mechanism of action. Molecular docking has further confirmed the possibility of its components to the targets.

**Results:**

As a result, 193 common targets between 1684 Danshen-related DEGs and 1492 UC targets were determined as the potential targets for Danshen in treatment with UC. Serum pharmacochemistry and target prediction showed that 22 components in serum acted on 777 targets. Intersection with common targets yielded 46 core targets, and an active ingredient-target-biological functional module network was constructed for analysis. Network prediction and molecular docking results showed that the main action modules were inflammatory response and cell apoptosis, which mainly acted on targets SRC, RELA, HSP90AA1, CTNNB1, STAT3, and CASP3. The main components of Danshen intervention in UC were predicted to include Catechol, 3,9-Dimethoxypterocarpan, 8-Prenylnaringenin, Isoferulic acid, Salvianolic acid C, and Danshensu.

**Discussion:**

This work resolved the ambiguity of Danshen’s anti-UC material basis and mechanism: 22 serum-absorbed components acted on 46 core targets to modulate inflammation and apoptosis, validating the integrated approach and laying groundwork for UC treatment and TCM research.

**Conclusion:**

The present study provides a scientific foundation for further explicating the mechanisms of Danshen against UC.

## 1 INTRODUCTION

Ulcerative colitis (UC) is a refractory recurrent gastrointestinal disease that primarily affects the colorectum's mucosal and submucosal layers. It has a high acute fulminant morbidity and mortality rate, a high cancer rate, a chronic persistent phenotype, and a proclivity for recurrence [1, 2]. UC has become a global disease, with an accelerated increase in incidence, especially in Asian countries [3]. The pathogenesis of UC is inconclusive, and epithelial barrier defects, genetic susceptibility, and the dysregulated immune response are generally considered possible pathogenic factors [4]. UC has been predominantly treated with sulfasalazine, mesalamine, and ustekinumab. Despite moderate therapeutic effects, some of these drugs may have adverse effects that increase the risk of neoplasia and infection [5]. Therefore, the search for therapeutic drugs for UC has become a hot research topic.

Traditional Chinese medicine (TCM) effectively treats UC with evidence-based treatment and minor adverse effects [6]. Currently, many effectiveness of TCM treatments have been proven clinically by non-controlled clinical trials and often utilised in the clinical treatment of UC are numerous Chinese patent medicines [7-11]. Danshen, the dried rhizome of *Salvia miltiorrhiza Bge*, first published in Shennong Ben Cao Jing, has a root resembling ginseng, with purple skin and purple flesh, hence the name Danshen. It works by activating blood circulation, dispelling blood stasis, cooling blood and eliminating carbuncles, clearing the heart and removing irritation, nourishing the blood, calming the mind, regulating menstruation, and relieving pain. Recently, evidence shows that Danshen has anti-inflammatory, antioxidant, and antibacterial properties and is effective in immunomodulation and intestinal epithelial protection [12, 13]. Moreover, it is reported that Danshen water extracts can significantly treat UC [14, 15] and promise UC treatment. However, the material basis is not yet comprehensively and systematically examined, and the mechanism of action remains ambiguous.

Studying the material basis and mechanism of action has been a crucial scientific issue in TCM modernization. As a new paradigm in drug research, network pharmacology systematically and comprehensively observes drug interventions and effects on disease networks based on the integration of multidisciplinary knowledge. It carries on the network analysis of the biological system, which coincides with the TCM concept of a holistic view and discriminatory treatment [16]. The approach of TCM network pharmacology includes network-based disease gene prediction, drug target and drug function, disease-specific network construction, herbal network construction, and quantitative drug-gene-disease co-module analysis [17]. This method can reflect herbal medicines' multi-component, multi-target, and multi-pathway action properties.

Network pharmacology is advantageous in explaining the synergistic effects of herbal medicines with components and targets. However, many components in herbal medicines that could not be absorbed are taken into account, while some absorbed components are excluded due to low absorption and other reasons. Therefore, serum medicinal chemistry is well suited to address this deficiency. In this study, UPLC-MS was used to analyze the absorbed prototype components and their metabolites and predict the targets of the components in serum to establish the herb-component-target relationship. In addition, to resolve the difficulty of guaranteeing the integrity of computational predictions alone, a herb target library was created using gene expression profiling for subsequent network pharmacological analysis.

Therefore, this study aimed to clarify the mechanism and potential bioactive components of Danshen treating UC by using comprehensive approaches based on serum pharmacochemistry, gene expression profiling, and network pharmacology analysis, to provide a theoretical basis for Danshen to treat UC. The workflow is shown in Fig. (**1**).

## 2 MATERIALS AND METHODS

### 2.1 UPLC-MS /MS Analysis of DANSHEN

#### 2.1.1 Preparation of Danshen Extract

Danshen was supplied by Qingdao Tiancheng Chinese Medicine Beverage Co. (Shandong, China) and verified by Prof. Li Baoguo of the Shandong University of TCM.

After soaking Danshen for one hour, we used 10 times as much water for the first extraction, boiling it for two hours and filtering it. Then we added 8 times the amount of water for the second extraction, boiling it for one hour, and combined both extracts. The filtrate was concentrated using a rotary evaporator and then formed into powder by a freeze-drying machine. The powder was dissolved in distilled water and used for drug administration.

#### 2.1.2 Animal Experiment

Six SD rats (200±20 *g*) were obtained from the Beijing Weitonglihua company (SCXK (Beijing) 2016-0006). They were raised in an environment with a temperature of 21~25°C and relative humidity of 50%~60%. The light and dark were alternating for 12 hours, and the noise was < 50 dB7. This study was approved by the Institutional Animal Care and Use Committee of Shandong University of Traditional Chinese Medicine (SDUTCM20220525001).

After adapting for 7 days, their blood was collected as blank control and then were given Danshen extract by gavage. The dosage of the Danshen extract is 45 mg/200 g which was chosen based on the adult daily dose of the crude drug (15 g/70 kg) according to the usage amount described in *Chinese pharmacopoeia*. The blood was obtained from the saphenous vein at 0 min, 5 min, 15 min, 30 min, 60 min, 90 min, and 120 min after administration. The supernatant was centrifuged to prepare serum samples, and the serum collected at different time points was mixed for analysis by UPLC/MS.

#### 2.1.3 Metabolites Extraction

After centrifuging the samples at 12000 rpm for 15 minutes at 4°C, 300 μL of the supernatant was transferred to a separate tube, and 1000 μL of the extracted solution containing 10 μg/mL of an internal standard was added. The samples were sonicated for five minutes in an ice-water bath. The samples were centrifuged for 15 minutes at 4°C after being set at -40°C for 1 hour (12000 rpm). Each sample's supernatant was meticulously filtered through a 0.22 μm microporous membrane, and 200 μL were pooled as QC samples.

Taken 400 μL of plasma sample, added 40 μL of hydrochloric acid (2 mol/L), vortexed for 1 min, and then set at 4°C for 15 min, and repeated four times. 1.6 mL acetonitrile was added, the mixture was vortexed for 5 min, and the samples were centrifuged at 12000 *rpm* for 5 min (4°C); then, the supernatant was dried by nitrogen. By vortex for 5 min, the dried samples were reconstituted with 80% methyl alcohol (10 μg/mL of the internal standard), then centrifuged at 12000 *rpm* for 5 min at 4°C. The supernatant was transferred to a new glass vial for LC/MS analysis.

#### 2.1.4 UPLC-MS

On an Agilent ultra-high performance liquid chromatography 1290 UPLC system with a Waters UPLC BEH C18 column (1.7 μm, 2.1×100 mm), UPLC-MS/MS analysis was carried out. A 5 μL injection volume was in use. 0.1% formic acid in water (A) and 0.1% formic acid in acetonitrile made up the mobile phase (B). The multi-step linear elution gradient program is shown in Table **1**.

Using a Q Exactive Focus mass spectrometer and Xcalibur software based on the IDA acquisition mode, MS and MS/MS data were collected. The detailed parameters are shown in the Table **2**.

### 2.2 Establishment of Target Database

#### 2.2.1 Screening of Disease Targets

Using “ulcerative colitis” as a search keyword, we searched the GeneCards database (https://www.GeneCards.org/) [18], DisGeNet database (https://www.disgenet.org/) [19], Drugbank database (https://go.drugbank.com/) [20], Therapeutic Target Database(http://db.idrblab.net/ttd/) and OMIM database (https://www.omim.org) [21] for UC-related targets. Genes with a score greater than 2.17 were screened using the GeneCards database; the DisGeNet database was used to inquire genes from the CTD-human database [22]; OMIM and TTD were used to collect UC-related data genes; Drugbank was applied to query the genes of UC therapeutic drug action. The UC target database was established by merging data from 5 databases and deleting duplicate or invalid genes.

#### 2.2.2 Gene Expression Profile

All mice were maintained in a temperature-controlled environment (22°C) with a 12 h light/12 h dark cycle and free access to water and food. Mice were divided into two groups at random: the control group, which was given waters orally, and the Danshen group, which was intragastrically administered with Danshen aqueous extract at 6.5 mg/20 g/day for 7 days. The dosage of the Danshen extract was chosen based on the adult daily dose of the crude drug (15 g/70 kg) according to the usage amount described in *Chinese pharmacopoeia*. CO_2_ executed mice after 7 days of dosing. The Trizol method isolated total RNA from liver tissues (n=3 samples per group). Sequencing libraries preparation and detection were used an Illumina RNALib preparation 2018, Novaseq 100 cycles 200M reads (20Gb), Illumina Novaseq SBS DMX Prep 200 Million Reads.R, illumina NGS Bioinformatics data package was applied for data analysis. A volcano map was used to show all differential genes through R packages of ggplot2.

### 2.3 PPI Network Construction

193 potential targets of Danshen were intersected with the targets related to UC, and a Venn diagram was created using the Venn diagram mapping website. We used the STRING database (https://string-db.org/) to construct a protein-protein interaction network for the Danshen Gene expression profile and UC intersection targets, setting the biological species as Homo sapiens with confidence level ≥ 0.400.

### 2.4 Gene Ontology (GO) Enrichment and KEGG Pathway Analyses

The common targets of Danshen in UC were imported into the DAVID Knowledgebase v2023q2 [23], for GO enrichment analysis and KEGG pathway enrichment analysis. The species selection “Homo sapiens”, and the relevant biological processes and pathways (*p* <0.05) were analyzed and visualized using the ggplot2 packages in Rstudio.

### 2.5 Target Prediction

Referring to the method of Fu [24] for target prediction, the components from Danshen in rat plasma were subjected to a target prediction algorithm comprising Bernoulli Naive Bayes profiling. More than 195 million bioactivity data points stored in the PubChem and ChEMBL libraries were assimilated to create this model. Targets were annotated using a full set of pathways from NCBI BioSystems and predicted on a per-compound base. For each component, we selected the targets with a score greater than 0.9 as the associated targets for subsequent analysis.

### 2.6 Active Ingredient-target-biological Functional Module Network Construction

The active ingredient-target-biological functional module network indicates the relationship between the active ingredients, targets, and functional biological modules. The network was analyzed by degree centrality analysis to investigate the mechanism of multiple components, targets, and pathways of Danshen in UC treatment.

The enrichment analysis results were annotated and classified to obtain the main biological functional module of Danshen for UC. The targets associated with each functional biological module were STRING (https://string-db.org/) [25]. The whole network, which combined the relationships between active ingredients and targets predicted, was imported into Cytoscape 3.9.1 [26].

### 2.7 Molecular Docking

For precise docking and analysis of the critical targets and main components of Danshen, molecular docking was done using CDOCK in the Receptor-Ligand International module of the programme Discovery Studio 2019 R2 [27]. According to their PubChem ID, the small molecule components of Danshen’s key active ingredients were obtained from PubChem (https://pubchem.ncbi.nlm.nih.gov/) and imported into Discovery Studio 2019 R2. By collecting the high-resolution crystal structures of the targets from the PDB [28], the active sites of the proteins are focused on the active amino acid sites that are labeled in the crystal structure itself for proligand action, constructing “Grid box “. This allows the system to search for “Grid box” near the active site. The “active pocket” information is located in the target “Grid box”. CDOCKER algorithm [29] module parameters are set: Pose Cluster Radius is 0.5, Random Conformations is 10, Orientations to Refine is 10, and the others default parameters keeps unchanged.

## 3 RESULTS

### 3.1 Candidate Targets Database of Danshen for UC

1728 disease targets were collected from the GeneCards database, 1258 from DisGeNet database, 110 from the OMIM database, 50 from TTD, and 82 from the Drugbank database. A total of 1728 UC-related targets were obtained after removing duplicates.

**Fig. (2):**
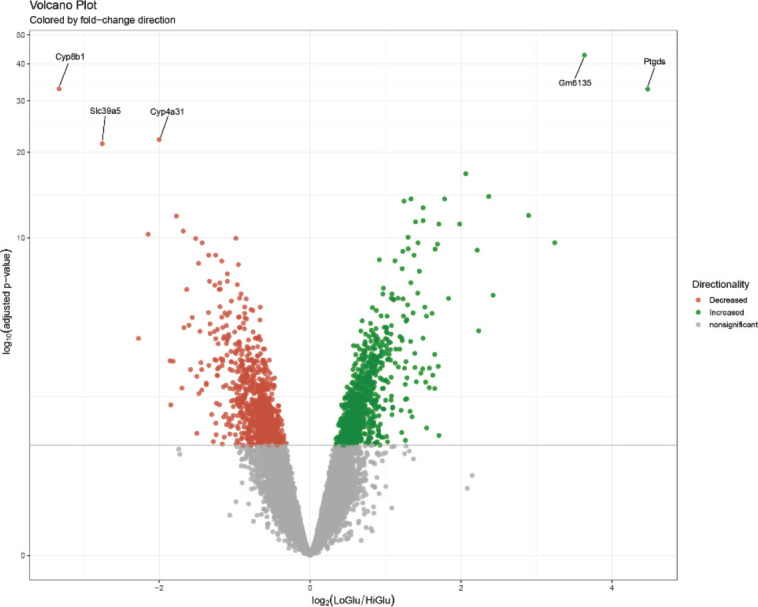
Volcano plot showing differentially expressed genes (DEG) in mice with Danshen. The upregulated genes (green), and the down-regulated genes (red) with *p* <0.05.

The differentially expressed genes (DEG) after Danshen treatment were analyzed by gene expression profile to find the possible targets of Danshen. *P*<0.05 were used as the criteria of DEG, and 1684 differential genes were obtained in the Danshen treated compared with the control group. There were 799 genes upregulated and 885 genes downregulated compared with the control group (Fig. **2**). As shown in the Fig. (**2**), Cyp8b1 (*x*= -3.33, *y*= 2.148755E-37), Slc39a5 (*x*= -2.75, *y=* 1.76E-25), Cyp4a31 (*x*= -2.00, *y=*2.70E-26), Gm6135 (*x*= 3.24, *y=* 5.10E-13) and Ptgds (*x*= 4.47, *y=* 3.78E-37) were significantly affected by Danshen and became important parts of the Danshen-related DEGs database. Gene expression profile of 193 common targets is shown in Table **S1**.

### 3.2 PPI Network

**Fig. (3):**
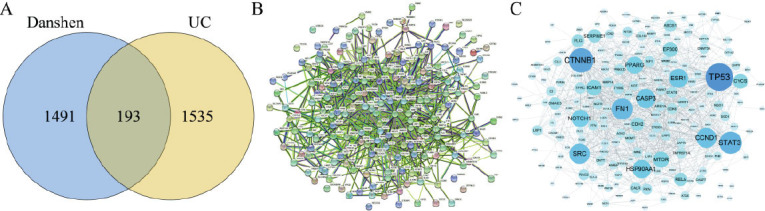
(**A**) Venn diagram of the drug-disease common target of Danshen and ulcerative colitis. (**B**) PPI network diagram of common targets from STRING. (**C**) PPI network diagram of common targets from Cytoscape.

The common targets between the Danshen-related DEGs and the targets of the UC were obtained by Venn analysis, and 193 common targets were determined as the potential targets for Danshen in treatment with UC (Fig. **3A**).

The potential targets were inputted into the STRING database to obtain the target protein interaction information and construct a PPI network diagram with 193 nodes (target proteins) and 1134 edges (protein interactions) (Fig. **3B**). Imported into Cytoscape for calculation and beautification. (Fig. **3C**). Node size and color show the magnitude of the degree value of this node. The larger the node and the darker color, the higher the corresponding degree value, indicating that more targets can effectively interact with this target among the common targets.

### 3.3 Enrichment Analysis

**Fig. (4):**
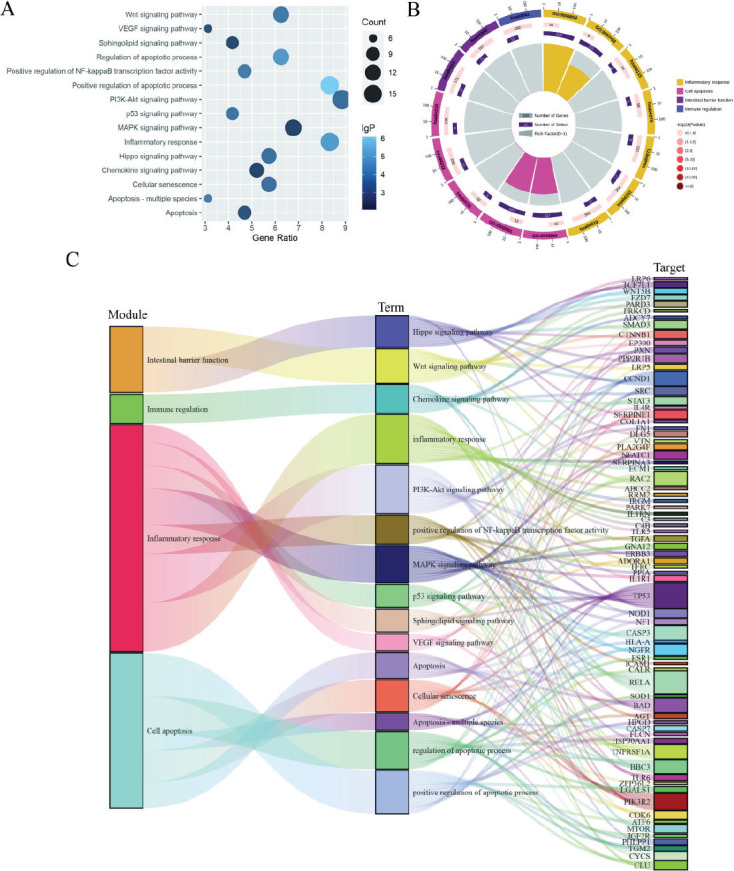
(**A**) Bubble plot of enrichment analysis of Danshen in treatment with UC. The gene ratio represents the proportion of targets in 193 common targets, bubble size depends on the target number of GO terms or pathways, and P-value determines the color (black represents a low value, and blue represents a high value). (**B**) Enrich circle of enrichment analysis of Danshen in treatment with UC. The first circle: Different colors represent different modules; The second circle is the classification number in the background gene and P-value. The more genes, the longer the bar, and the smaller the value, the redder the color; The third circle: the bar chart of foreground gene ratio; The fourth circle: RichFactor value of each term (the number of foreground genes divided by the number of background genes in this classification). (**C**) Sankey diagram of enrichment analysis of Danshen in treatment with UC. It shows the relationship and data flow between modules (left), enrichment entries (middle), and targets (right).

The 193 common targets were entered into the DAVID Knowledgebase v2023q2 for GO and KEGG pathway enrichment analysis, and the results showed 345 biological processes, 83 molecular functions, 86 cellular components, and 120 pathways. The top 15 entries of the above four categories were visualized by filtering by *p* <0.05 and a high number of target enrichment (Fig. **4** and Table **3**). These terms mainly focused on inflammatory response and cell apoptosis modules, which shows Danshen may affect the UC process mainly through these two modules.

### 3.4 Analysis of Danshen in the Blood Serum

Danshen extract, the serum of Danshen-treated mice, and blank serum was analyzed by UPLC-MS (Fig. **S1**). The retention time, UV, and MS spectra were compared with those from reference samples. 22 main components/metabolites were detected qualitatively in the serum of Danshen-treated mice (Table **4**). These blood components are mainly phenylpropanoids, flavonoids, alkaloids, and their derivatives, which exerted anti-inflammatory and cell apoptosis effects [30-32]. Since most of the drug components are absorbed into the blood circulation and exert their effects, the above 22 components may be the potential active ingredients of Danshen for the treatment of UC.

### 3.5 Target Prediction and Component – Target Interaction Network

**Fig. (5):**
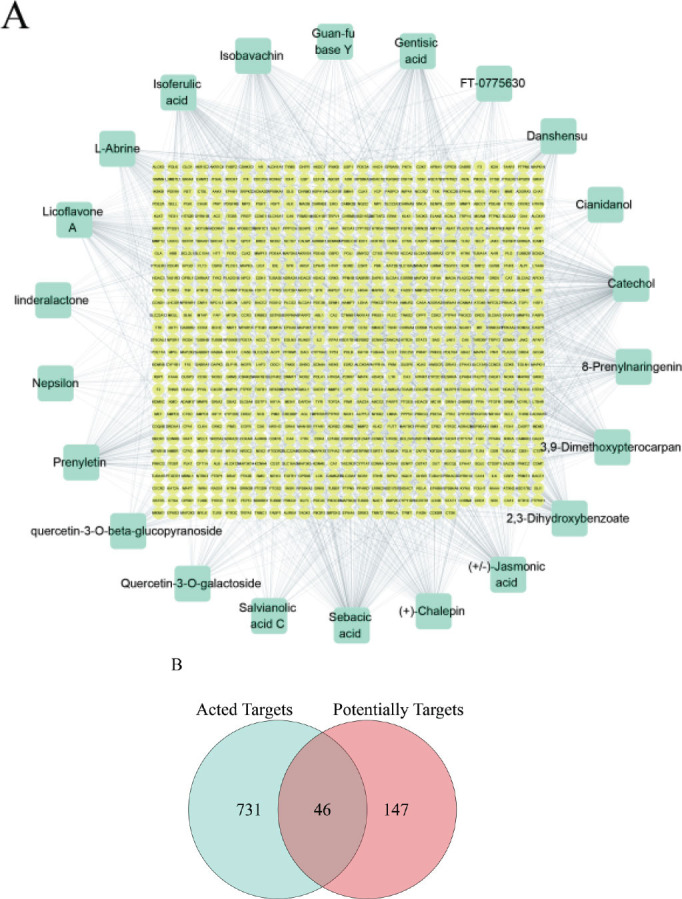
(**A**) Danshen - component - target interaction network, yellow circles are targets, blue squares are components in serum. (**B**) Venn diagram of the common target of 193 potential targets and 777 targets acted by 22 components in serum.

Using the prediction method above, we obtained the relationship between components in serum and targets. The results showed that 22 components acted on 777 targets. The active components in serum and predicted target relationships were imported into Cytoscape-3.9.1 to construct the active ingredient-target network (Fig. **5A**). A venn diagram was created by the database of common targets (potential targets) and Danshen constituent targets to get 46 core targets used for post-analysis and network construction (Table **5** and Fig. **5B**).

### 3.6 Active Ingredient-target-Biological Functional Module Network

**Fig. (6):**
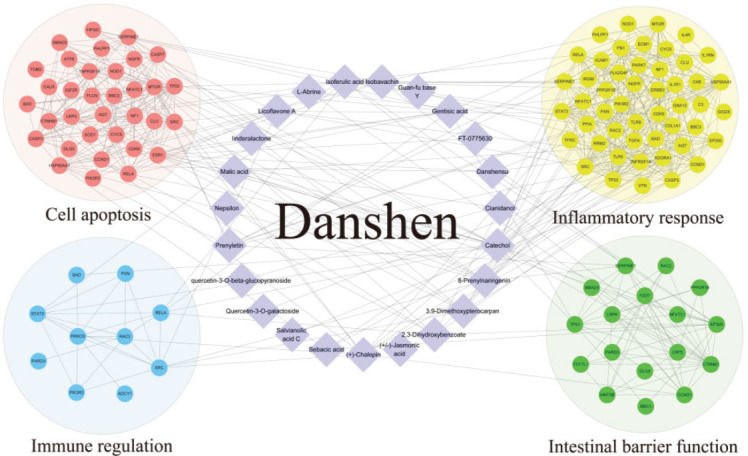
Active ingredient-target-biological functional module network of Danshen for treating UC. The lines between the nodes stand for the predicted relationship of active ingredient-target and target-target. Purple rhombus represents the active ingredient in the blood serum; Blue, red, green, and yellow round stands for targets in a different module.

After importing the relationship between components in serum and targets and the relationship between targets in each biological function module into the network, the active ingredient-target-biological functional module network is completed (Fig. **6**). By calculating the degree in the network, we found that the inflammatory response and cell apoptosis modules are the main modules in the network. SRC, TP53, RELA, HSP90AA1, CASP3 are the key targets. The key components are catechol, Gentisic acid, 3,9-Dimethoxypterocarpan, 8-Prenylnaringenin, and Isoferulic acid. Those results elucidate the rules of using Danshen for treating UC from a molecular network perspective. Danshen multi-component acts on multiple targets and modules, reflecting the multi-component, multi-target, and multi-module coordinated and co-regulatory action characteristics of Danshen for UC.

### 3.7 Molecular Docking

The docking of targets and their related components in the network revealed negative docking energies, indicating that they both dock well (Table **6**). EP300 had the lowest docking CDOCKER energy of -61.0874 with Sebacic acid, EP300 and Malic acid, EP300 and 2,3-Dihydroxybenzoate CASP3 and Salvianolic acid C, EP300 and Danshensu all had docking energies <-40 kcal/mol. The docking patterns are shown in Fig. (**7**).

## 4 DISCUSSION

**Fig. (8):**
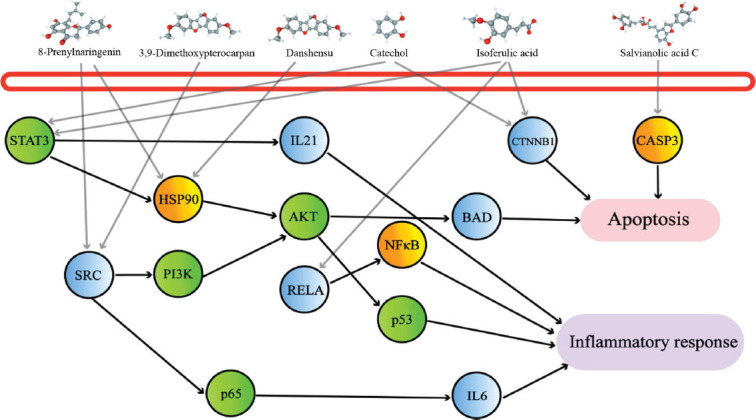
The possible mechanism used by Danshen to UC.

Danshen has the effect of cooling blood and eliminating carbuncles. It has been used to treat sores and pains, hypochondriac and abdominal pains, and obstruction and accumulation in the abdomen in Chinese medicine. Its efficacy and indications fit the basic pathogenesis of UC. In this study, containing components, targets, and pathways were constructed to explore the potential mechanism of action of Danshen in the treatment of UC (Fig. **8**).

After oral administration of most TCM, drug effects may occur only when the components migrate into the blood and interact with the body. The UPLC-MS method was employed to analyze the components of Danshen in plasma and performed target prediction to get the component-target relationship containing 22 components acting on 777 targets. Meanwhile, through differentially expressed genes (DEG) and database mining, we identified 193 possible targets of Danshen for UC and conducted the enrichment analysis to link and classify the targets. After importing the relationship between components in serum and targets and the relationship between targets in each biological function module into the network, a network system containing components, targets, and biological functional modules was constructed to analyze and visualize the mechanism of Danshen in the treatment of UC.

The network diagram shows that Danshen for UC mainly acts through two functional biological modules: cell apoptosis and inflammatory response. Apoptosis is essential for intestinal tissue homeostasis and intestinal epithelial cell renewal. Excessive apoptosis is linked to the inflammatory status of UC [33]. Previous studies have also shown that inhibiting apoptosis can prevent DSS-induced UC [34]. The targets SRC, RELA, HSP90AA1, and CTNNB1, are at the core of the network in the apoptosis module. Previous research found that Src kinase phosphorylated caspase-9 to increase its apoptotic activity, and caspase-9 aggregation and activation can induce DNA damage and cell death [35, 36]. Wnt and Src-YAP signals act together induce intestinal regeneration and intestinal apoptosis [37]. RELA, also known as the NF-B p65 subunit, is essential for the homeostatic control of intestinal epithelia cell death and division while also guarding against the development of acute, severe gastrointestinal inflammation [38]. HSP90 increases the growth of certain cells by potentiating glycolysis and proliferation while reducing apoptosis through PKM2 [39]. CTNNB1 encodes catenin beta 1 protein and has been widely described as a crucial molecule in Wnt/β-catenin signaling [40]. CTNNB1 is extensively proposed to promote cell proliferation and suppress cell apoptosis under ox-LDL treatment [41]. The molecular docking results showed that Catechol, 3,9-Dimethoxypterocarpan, 8-Prenylnaringenin, and Isoferulic acid could better act on SRC, RELA, HSP90AA1, and CTNNB1. It has been shown that these components can affect UC by acting on the apoptosis module. Isoferulic acid affects rat pancreatic β-cell apoptosis through the mitochondrial survival pathway [42]. Catechol can induce DNA damage and apoptosis to affect cancer progression [43]. 8-Prenylnaringenin inhibits the proliferation of HCT-116 colon cancer cells by inducing apoptosis through both intrinsic and extrinsic pathways [44].

UC is affected by multiple inflammatory pathways and is associated with various inflammatory factors. Serum TNF-α and IL-6 levels are significantly higher in patients with UC than in the healthy population and are positively connected with the severity of the disease. IL-6 is a critica factor in the development of colonic inflammation, causing intestinal inflammation and inducing inflammation toward tumorigenesis [45]. Danshen can reduce serum HIF-1α, COX-2, TNF-α, and IL-6 levels while increase IL-10 levels, which affect the inflammatory response [46]. There are extractive components in Danshen that could constrict damage in colon which induced by DSS and overexpression of pro-inflammatory cytokines *via* the RIPs-MLKL-Caspase-8 axis [47]. SRC, STAT3, HSP90AA1, and CASP3 have the dominant degree in the network and are the main targets of inflammatory response. SRC binds to and activates PIK3R1 (p85), a PI3K signaling driver, causing the NF-κB signalling cascade to be activated, contributing to persistent inflammatory responses in damaged intestinal tissues [48]. Studies have shown that inhibition of STAT3 leads to increased inflammatory response, explicitly affecting pathways regarding JAK/STAT [49]. The molecular docking results showed that Salvianolic acid C, 3,9-Dimethoxypterocarpan, 8-Prenylnaringenin, and Danshensu could better act on SRC, TP53, STAT3, HSP90AA1, and CASP3. Salvianolic acid C reversed the LPS-induced inflammatory response, inhibited NF-κB activation, and upregulated the expression of p-AMPK, HO-1, Nrf2 and NQO1 [50]. Danshensu affected the NF-B signalling pathway, inhibited IL-1-induced phosphorylation of p-I-B and p-p65, and decreased COX-2 and iNOS protein expression in IL-1-treated cells, decreasing the inflammatory response [51].

## STUDY LIMITATIONS

While this research advances understanding of Danshen’s role in UC treatment, some elements could be expanded to boost its robustness. For core targets like RELA and HSP90AA1, molecular docking has laid a foundation for their interaction with Danshen components, but follow-up experiments would be valuable to confirm such modulation in UC-specific tissues, deepening support for the proposed mechanisms. The network pharmacology framework, which leverages database targets and predictions, could also benefit from validation-testing how perturbing key nodes affects UC phenotypes would reinforce the network’s relevance. Furthermore, specific recommendations for clinical application are yet to be fully addressed, which would aid in translating these preclinical findings to practical therapy.

## CONCLUSION

In summary, we've developed a comprehensive system integrating serum medicinal chemistry, gene expression profiling, and network pharmacology to uncover Danshen's complex actions and active ingredients for UC. Key active components like Catechol, 3,9-Dimethoxypterocarpan, 8-Prenylnaringenin, Isoferulic acid, Salvianolic acid C, and Danshensu may alleviate UC by targeting SRC, RELA, HSP90AA1, CTNNB1, STAT3, and CASP3, affecting apoptosis and inflammatory response. Our results reflect the characteristics of the joint action of multiple components of TCM. It provides a basis for further mechanistic studies and makes subsequent studies more relevant.

## KEY MESSAGES

- The differentially expressed genes after Danshen treatment were analyzed by gene expression profile to find the possible targets of Danshen. Based on serum pharmacochemistry, we obtained the ingredients of Danshen in serum.
- Through network pharmacology analysis we get the active ingredients of Danshen, such as Catechol, 3,9-Dimethoxypterocarpan, 8-Prenyl-naringenin, Isoferulic acid, Salvianolic acid C, and Danshensu, those that might ameliorate UC by acting on SRC, RELA, HSP90AA1, CTNNB1, STAT3, and CASP3, which partly affected cell apoptosis and inflammatory response.
- A novel integrated research approach for exploring the substance basis and mechanism of TCM in treating diseases, including serum pharmacochemistry, gene expression profiling, network Pharmacology and molecular docking.

## Figures and Tables

**Fig. (1):**
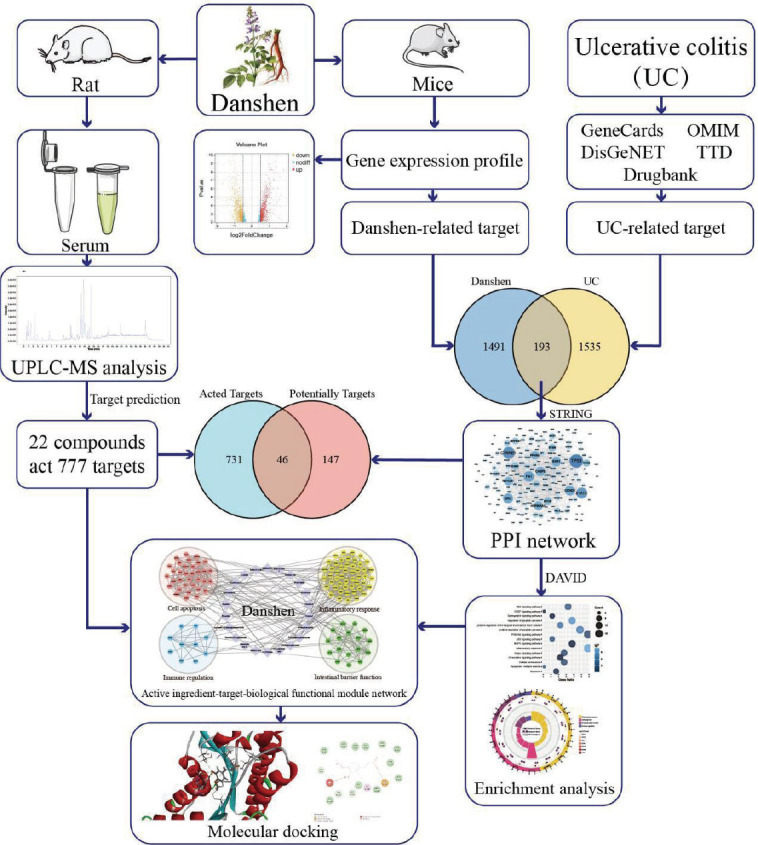
Workflow of exploration of mechanism of Danshen in the treatment of UC.

**Fig. (7):**
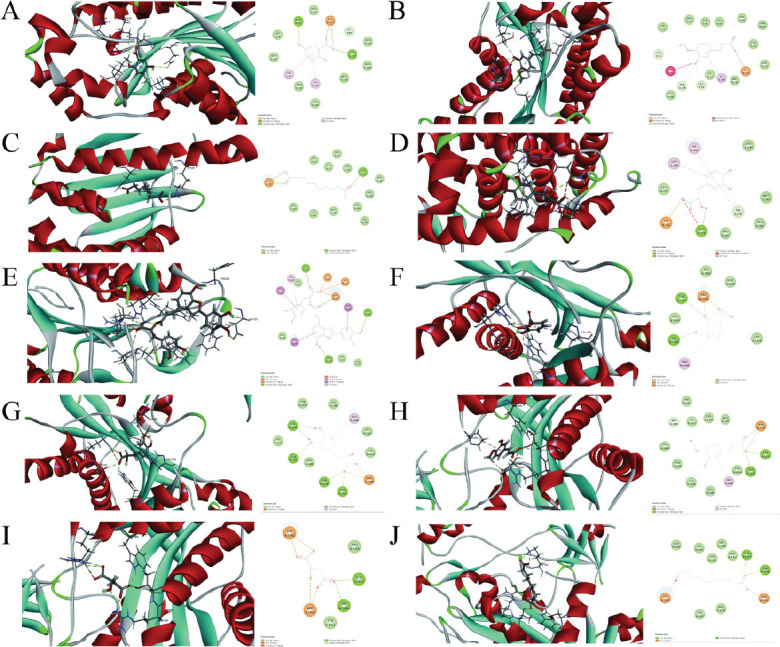
3D and 2D images of interaction between targets and compounds for Danshen in treatment with UC. (**A**) 2GDZ and Gentisic acid; (**B**) 2GDZ and Isoferulic acid; (**C**) 3WQ9 and Sebacic acid; (**D**) 4ZNH and Danshensu; (**E**) 5I9B and Salvianolic acid C; (**F**) 5LKZ and 2,3-Dihydroxybenzoate; (**G**) 5LKZ and Danshensu; (**H**) 5LKZ and Isoferulic acid; (**I**) 5LKZ and Malic acid and (**J**) 5LKZ and Sebacic acid.

**Table 1:** The multi-steplinear elution gradient program.

**Time (min)**	**Flow Rate (mL/min)**	**A%**	**B%**
0	400	95	5
3.5	400	85	15
6	400	70	30
6.5	400	70	30
12	400	30	70
12.5	400	30	70
18	400	0	100
22	400	0	100
25	400	0	100
26	400	95	5
30	400	95	5

**Table 2:** MS condition.

**Term**	**Parameter**
Capillary temperature	400°C
Full ms resolution	70000
MS/MS resolution	17500
Sheath gas flow rate	45 Arb
Aux gas flow rate	15 Arb
Collision energy	15/30/45 in NCE mode
Spray Voltage	4.0 kV (positive) or -3.6 kV (negative)

**Table 3:** Enriched GO biological processes or KEGG signaling pathways of common targets.

**Module**	**Type**	**Term**	***p*-value**
Inflammatory response	KEGG	hsa04115: p53 signaling pathway	3.59×10-04
-	KEGG	hsa04151: PI3K-Akt signaling pathway	6.40×10-04
-	KEGG	hsa04370: VEGF signaling pathway	4.13×10-03
-	KEGG	hsa04071: Sphingolipid signaling pathway	6.22×10-03
-	KEGG	hsa04010: MAPK signaling pathway	8.47×10-03
-	GO(BP)	GO:0006954: inflammatory response	2.17×10-05
-	GO(BP)	GO:0051092: positive regulation of NF-kappaB transcription factor activity	2.25×10-04
Immune regulation	KEGG	hsa04062: Chemokine signaling pathway	7.95×10-03
Intestinal barrier function	KEGG	hsa04310: Wnt signaling pathway	2.59×10-04
-	KEGG	hsa04390: Hippo signaling pathway	5.35×10-04
Cell apoptosis	KEGG	hsa04215: Apoptosis - multiple species	2.45×10-04
-	KEGG	hsa04218: Cellular senescence	5.10×10-04
-	KEGG	hsa04210: Apoptosis	3.17×10-03
-	GO(BP)	GO:0043065: positive regulation of apoptotic process	8.37×10-07
-	GO(BP)	GO:0042981: regulation of apoptotic process	2.86×10-05

**Table 4:** 22 components/metabolite found in the serum of Danshen by UPLC-MS compared to the model group.

**Component Name**	**Rt (min)**	**Quasi. (m/z)**	**Cal. (m/z)**	**Ppm**	**Ion Mode**
Nepsilon	0.62	189.1593	188.27	1.4580	Negative
2,3-Dihydroxybenzoate	1.55	153.0192	154.12	1.1790	Negative
Danshensu	1.6	197.0454	198.17	2.2221	Negative
Catechol	1.86	109.0294	110.11	3.6807	Negative
L-Abrine	2.47	219.1118	218.25	0.8599	Positive
Gentisic acid	2.76	153.0191	154.12	0.7407	Negative
Cianidanol	3.87	291.0832	290.27	6.2441	Positive
Quercetin-3-O-beta-glucopyranoside	5.29	465.1014	464.4	0.8959	Positive
Quercetin-3-O-galactoside	5.32	463.0882	464.4	0.4213	Negative
Sebacic acid	5.66	201.1129	202.25	0.3328	Negative
Isoferulic acid	6.86	195.0646	194.18	2.2833	Positive
3,9-Dimethoxypterocarpan	7.2	283.0970	284.31	0.0052	Negative
Salvianolic acid C	7.82	493.1107	492.4	0.5185	Positive
(+/-)-Jasmonic acid	8.13	211.1323	210.27	1.2567	Positive
Linderalactone	8.37	245.1165	244.28	2.0536	Positive
Prenyletin	9.13	269.0772	246.26	0.6185	Positive
Isobavachin	9.61	325.1398	324.4	0.7638	Positive
Licoflavone A	9.92	323.1240	322.4	0.1171	Positive
8-Prenylnaringenin	9.96	341.1365	340.4	1.6004	Positive
FT-0775630	10.01	357.1336	358.4	1.1301	Negative
Guan-fu base Y	11.48	388.2137	387.5	9.5373	Positive
(+)-Chalepin	11.61	337.1397	314.4	0.8367	Positive

**Table 5:** 46 core target information in PPI network, component - target interaction network, and Gene expression profile.

**Target**	**Degree**	**Betweeness**	**Closeness**	***p*-value**	**log2 Fold Change**	**Act Components**
TP53	80	0.15	0.62	5.48×10^-04^	-0.72	2
CTNNB1	68	0.11	0.58	7.23×10^-03^	-0.36	3
STAT3	61	0.07	0.57	1.18×10^-04^	-0.68	2
SRC	53	0.05	0.55	2.48×10^-03^	-0.96	4
CCND1	52	0.04	0.54	1.45×10^-11^	0.92	1
CASP3	49	0.03	0.55	3.75×10^-04^	0.59	2
ESR1	49	0.06	0.55	1.40×10^-04^	0.62	9
HSP90AA1	47	0.06	0.54	4.45×10^-03^	-0.42	6
PPARG	42	0.05	0.53	3.44×10^-04^	-0.71	8
MTOR	38	0.04	0.52	2.53×10^-05^	-0.67	4
ICAM1	36	0.03	0.51	1.11×10^-03^	-0.77	1
EP300	34	0.02	0.5	1.44×10^-03^	-0.62	5
SERPINE1	30	0.02	0.49	1.94×10^-03^	-1.25	3
RELA	29	0.01	0.49	1.86×10^-03^	-0.54	4
PLG	27	0.04	0.49	8.85×10^-05^	-0.52	3
ABCB1	25	0.04	0.49	3.02×10^-09^	1.09	9
SMAD3	25	0.02	0.49	7.47×10^-03^	-0.62	1
TYMS	21	0.02	0.46	2.17×10^-03^	0.69	2
CDK6	19	0	0.47	2.96×10^-03^	-0.51	3
PRKCD	19	0	0.47	7.27×10^-03^	-0.59	1
ERBB3	18	0.01	0.48	1.36×10^-03^	-0.65	1
CASP7	16	0.01	0.46	1.70×10^-03^	0.46	1
MME	16	0	0.46	3.37×10^-07^	0.81	5
NGFR	16	0	0.45	2.02×10^-04^	-1.47	1
NQO1	16	0.02	0.44	2.50×10^-03^	0.65	1
ZAP70	15	0	0.46	9.47×10^-04^	0.74	1
ANPEP	13	0	0.44	3.48×10^-11^	-0.95	1
DHFR	12	0.01	0.44	1.12×10^-03^	0.61	2
CYP1A2	11	0.01	0.39	2.14×10^-03^	-0.37	3
NAMPT	11	0	0.43	9.07×10^-06^	0.84	1
ODC1	10	0	0.42	4.19×10^-03^	0.53	3
PPIA	10	0	0.41	3.46×10^-06^	0.71	3
TPMT	10	0	0.38	2.12×10^-03^	0.53	1
PLEC	9	0.01	0.41	8.40×10^-04^	-0.84	1
ARG1	8	0	0.41	5.63×10^-06^	0.68	1
BAD	7	0	0.41	1.91×10^-03^	0.6	1
PDE4A	7	0	0.38	3.93×10^-03^	-0.91	1
GUSB	6	0	0.41	5.23×10^-03^	-0.43	1
HSD11B1	5	0	0.37	1.50×10^-04^	0.57	6
PNP	5	0	0.33	5.01×10^-03^	0.47	1
RORC	5	0	0.39	6.49×10^-06^	0.83	2
BLM	4	0	0.39	1.41×10^-03^	0.82	14
ADORA1	3	0	0.33	1.18×10^-03^	-0.65	7
HPGD	2	0	0.35	1.39×10^-03^	0.58	5
SLC16A1	1	0	0.33	1.28×10^-03^	-0.59	2
ST6GAL1	1	0	0.28	9.33×10^-07^	-0.7	1

**Table 6:** Molecular docking details and results.

**Target**	**PDB ID**	**Component**	**CDOCKER Energy (kcal/mol)**
ADORA1	6D9H	Quercetin-3-O-beta-glucopyranoside	-13.5687
-	-	Isobavachin	-14.7561
-	-	8-Prenylnaringenin	-17.8016
-	-	Licoflavone A	-17.9814
-	-	Quercetin-3-O-galactoside	-13.5687
-	-	Prenyletin	-9.5934
CASP3	5I9B	Salvianolic acid C	-46.4981
CTNNB1	1G3J	Isoferulic acid	-0.295203
-	-	Catechol	-23.7382
EP300	5LKZ	2,3-Dihydroxybenzoate	-42.9538
-	-	Malic acid	-43.2717
-	-	Isoferulic acid	-38.1354
-	-	Danshensu	-45.7762
-	-	Catechol	-16.5204
-	-	Sebacic acid	-61.0874
ESR1	4ZNH	Danshensu	-34.6268
-	-	Catechol	-13.9441
-	-	Gentisic acid	-23.5071
-	-	Isobavachin	-7.04488
-	-	8-Prenylnaringenin	-5.78315
-	-	Licoflavone A	-18.9612
-	-	Cianidanol	-21.0969
HPGD	2GDZ	2,3-Dihydroxybenzoate	-30.0273
-	-	Catechol	-20.4782
-	-	Gentisic acid	-33.4082
-	-	Isoferulic acid	-34.7959
-	-	Licoflavone A	-32.2844
HSP90AA1	3WQ9	8-Prenylnaringenin	-16.596
-	-	Danshensu	-30.8533
-	-	Gentisic acid	-18.826
-	-	Isobavachin	-15.3556
-	-	Prenyletin	-8.29558
-	-	Sebacic acid	-37.9672
MTOR	5zcs	Isobavachin	-11.1902
-	-	8-Prenylnaringenin	-8.61854
-	-	Prenyletin	-2.89146
RELA	1NFI	2,3-Dihydroxybenzoate	-5.87279
-	-	Isoferulic acid	-15.443
-	-	Gentisic acid	-23.2195
SRC	1Y57	3,9-Dimethoxypterocarpan	-0.463539
-	-	8-Prenylnaringenin	-13.9542
-	-	Licoflavone A	-17.8499

## Data Availability

The original contributions presented in the study are included in the article/Supplementary Material, further inquiries can be directed to the corresponding authors.

## LIST OF ABBREVIATIONS

BP: Biological Process
CC: Cellular Component
DEG: Differentially Expressed Genes
DSS: Sodium Dextran Sulfat
GO: Gene Ontology
MF: Molecular Function
NF-κB: Nuclear Factor-kappa B
PPI: Protein-protein Interactions
TCM: Traditional Chinese Medicine
UC: Ulcerative Colitis
UPLC-MS: Ultra Performance Liquid Chromatography - Tandem Mass Spectrometer
