# Supplementary material for: Exploring the Potential Mechanisms of Danshen for the Treatment of Ulcerative Colitis based on Serum Pharmacochemistry, Gene Expression Profiling, and Network Pharmacology: Regulation of Cell Apoptosis and Inflammatory Response
Source: Curr Comput Aided Drug Des. 2024 Oct 10;22(1):25–42. doi: 10.2174/0115734099318174240926103444 (PMC13358774; doi:10.2174/0115734099318174240926103444)
Supplement: Supplementary file 1 [file CCADD-22-1-25_SD1.pdf]

SUPPLEMENTARY MATERIAL

Exploring the Potential Mechanisms of Danshen for the Treatment of Ulcerative Colitis based on Serum Pharmacochemistry, Gene Expression Profiling, and Network Pharmacology: Regulation of Cell Apoptosis and Inflammatory Response

Run-Xiang Zhai[1,2], Meng-Yu Wang[1,2,3], Hai-Tao Du[4], Chun-Xiao Yan[1,2], Zi-Wei Li[1,2], Kuo Xu[1,2], Hui Li[1,2], Xian-Jun Fu[1,2,*] and Xia Ren[1,2,*]

[1]Marine Traditional Chinese Medicine Research Center, Qingdao Academy of Traditional Chinese Medicine, Shandong University of Traditional Chinese Medicine, Qingdao, 266114, China; [2]Qingdao Key Technology Innovation Center of Marine Traditional Chinese Medicine Deep Development and Industrialization, Qingdao, 266114, China; [3]State Key Laboratory of Quality Research in Chinese Medicine, Macau University of Science and Technology, Taipa, Macau, China; [4]College of Pharmacy, Shandong University of Traditional Chinese Medicine, Jinan, 250355, China

Table S1. Gene expression profile information of common targets.

Gene Name (Mus Musculus)	Gene Name (Homo Sapiens)	log2FoldChange	*p*-value
Ptp4a1	PTP4A1	0.72	2.46×10-04
Dst	DST	-0.54	5.03×10-03
Zap70	ZAP70	0.74	9.47×10-04
Il1r1	IL1R1	0.73	5.23×10-04
Fzd7	FZD7	-0.69	7.09×10-03
Fn1	FN1	-0.93	1.61×10-06
Igfbp2	IGFBP2	0.75	4.95×10-05
Ugt1a1	UGT1A1	-0.53	3.42×10-05
Phlpp1	PHLPP1	-0.71	5.30×10-04
Adora1	ADORA1	-0.65	1.18×10-03
Cfhr1	CFHR1	0.76	5.09×10-05
Cfhr2	CFHR2	0.96	3.12×10-09
F5	F5	-0.69	1.81×10-05
Atf6	ATF6	-0.43	5.72×10-03
Tlr5	TLR5	-2.14	8.37×10-14
Hsd11b1	HSD11B1	0.57	1.50×10-04
Trdmt1	TRDMT1	0.86	4.01×10-04
Hnmt	HNMT	1.08	5.66×10-09
Il1rn	IL1RN	-0.90	4.16×10-04
Sec16a	SEC16A	-0.68	1.70×10-04
Notch1	NOTCH1	-1.06	1.33×10-04
Adamts13	ADAMTS13	-0.68	3.20×10-03

1875-6697/26 $65.00+.00 © 2026 Bentham Science Publishers

Lcn2	LCN2	-1.38	8.37×10-05
Gpd2	GPD2	-0.56	4.53×10-04
Ptprj	PTPRJ	-0.53	3.32×10-03
Pla2g4f	PLA2G4F	-1.43	1.56×10-04
Itpa	ITPA	0.75	3.44×10-04
Pcna	PCNA	0.74	1.68×10-05
Src	SRC	-0.96	2.48×10-03
Tgm2	TGM2	-0.85	6.44×10-09
Psma7	PSMA7	0.80	1.32×10-05
Mme	MME	0.81	3.37×10-07
Sema4a	SEMA4A	-0.78	5.15×10-04
Adar	ADAR	-0.61	5.66×10-04
S100a1	S100A1	0.86	2.79×10-06
Rorc	RORC	0.83	6.49×10-06
Ecm1	ECM1	-0.54	4.97×10-04
Hsd3b3	HSD3B1	0.66	2.97×10-04
Slc16a1	SLC16A1	-0.59	1.28×10-03
Bag1	BAG1	0.56	3.97×10-04
Abca1	ABCA1	-0.59	1.15×10-03
Lepr	LEPR	-0.52	7.59×10-03
Ak2	AK2	0.46	5.28×10-03
Arid1a	ARID1A	-0.95	2.00×10-05
Mtor	MTOR	-0.67	2.53×10-05
Tmem201	TMEM201	-0.69	1.59×10-03
Park7	PARK7	0.36	7.44×10-03
Cdk6	CDK6	-0.51	2.96×10-03
Abcb1a	ABCB1	1.09	3.02×10-09
Tyms	TYMS	0.69	2.17×10-03
Hps4	HPS4	-0.50	6.45×10-03
Pxn	PXN	-0.66	1.22×10-03
Hectd4	HECTD4	-0.78	7.32×10-04
Gusb	GUSB	-0.43	5.23×10-03
Cldn3	CLDN3	-0.51	5.88×10-05
Serpine1	SERPINE1	-1.25	1.94×10-03
Gna12	GNA12	-0.78	2.55×10-08
Cyp3a16	CYP3A5	-1.52	2.23×10-13
Agbl3	AGBL3	0.81	5.39×10-03
Cycs	CYCS	0.94	5.81×10-08
Nod1	NOD1	-0.58	1.91×10-03
Ggcx	GGCX	-0.87	4.89×10-05
Tcf7l1	TCF7L1	-0.89	1.35×10-03
Tgfa	TGFA	-0.54	7.86×10-03
Pparg	PPARG	-0.71	3.44×10-04
Wnt5b	WNT5B	1.03	8.83×10-06

Tnfrsf1a	TNFRSF1A	-0.51	2.63×10-03
Pzp	PZP	-0.88	1.44×10-07
Lrp6	LRP6	-0.46	3.01×10-03
Bbc3	BBC3	-0.72	5.41×10-03
Ceacam1	CEACAM1	-0.83	3.98×10-05
Anpep	ANPEP	-0.95	3.48×10-11
Furin	FURIN	-0.79	6.29×10-05
Blm	BLM	0.82	1.41×10-03
Lrrc32	LRRC32	-0.76	4.74×10-04
Il4ra	IL4R	-0.58	4.03×10-03
Sult1a1	SULT1A1	0.48	9.47×10-04
Mgmt	MGMT	0.47	5.24×10-03
Ccnd1	CCND1	0.92	1.45×10-11
Slc10a2	SLC10A2	2.21	2.48×10-12
Defb1	DEFB1	1.28	9.64×10-08
Casp3	CASP3	0.59	3.75×10-04
Hpgd	HPGD	0.58	1.39×10-03
Ndufa13	NDUFA13	0.45	2.52×10-03
Gdf15	GDF15	-1.15	1.24×10-03
Pik3r2	PIK3R2	-0.61	7.08×10-03
Myo9b	MYO9B	-0.63	5.21×10-04
Calr	CALR	-0.47	3.00×10-03
Adcy7	ADCY7	-0.79	3.03×10-03
Nqo1	NQO1	0.65	2.50×10-03
Agt	AGT	-0.66	1.89×10-08
Pard3	PARD3	-0.56	3.88×10-04
Icam1	ICAM1	-0.77	1.11×10-03
Pde4a	PDE4A	-0.91	3.93×10-03
Nlrx1	NLRX1	-0.70	7.66×10-03
Ppp2r1b	PPP2R1B	-1.10	4.47×10-10
Cyp1a2	CYP1A2	-0.37	2.14×10-03
Smad3	SMAD3	-0.62	7.47×10-03
Nt5e	NT5E	-0.69	1.42×10-04
Ctdspl	CTDSPL	-0.75	4.90×10-03
Ctnnb1	CTNNB1	-0.36	7.23×10-03
Cyp8b1	CYP8B1	-3.33	2.15×10-37
Esr1	ESR1	0.62	1.40×10-04
Syne1	SYNE1	-0.71	8.68×10-04
Arg1	ARG1	0.68	5.63×10-06
Atg5	ATG5	0.60	6.17×10-04
Cisd1	CISD1	0.42	4.96×10-04
Gstt1	GSTT1	0.50	2.73×10-04
Sbno2	SBNO2	-0.58	1.00×10-03
Tcf3	TCF3	-0.60	2.99×10-03

Lrp1	LRP1	-0.74	2.01×10-04
Stat6	STAT6	-0.41	2.21×10-03
Erbb3	ERBB3	-0.65	1.36×10-03
Ppia	PPIA	0.71	3.46×10-06
Ddc	DDC	-0.58	3.15×10-03
Sar1b	SAR1B	0.68	2.49×10-05
Irgm2	IRGM	-0.76	2.56×10-06
Flcn	FLCN	-0.61	4.67×10-04
Trp53	TP53	-0.72	5.48×10-04
Prpf8	PRPF8	-0.60	7.10×10-04
Vtn	VTN	-0.47	5.29×10-05
Nf1	NF1	-0.60	2.71×10-03
Ccl9	TLR6	1.30	1.58×10-13
Col1a1	COL1A1	-0.98	7.74×10-03
Ngfr	NGFR	-1.47	2.02×10-04
Phb	PHB	0.48	3.56×10-03
Lasp1	LASP1	-0.69	9.75×10-05
Jup	JUP	-0.78	7.07×10-05
Stat3	STAT3	-0.68	1.18×10-04
Lgals3bp	LGALS3BP	-0.87	1.82×10-08
Dnmt3a	DNMT3A	-0.66	1.39×10-03
Odc1	ODC1	0.53	4.19×10-03
Rrm2	RRM2	0.74	7.17×10-03
Nampt	NAMPT	0.84	9.07×10-06
Serpina3n	SERPINA3	-0.59	1.26×10-05
Hsp90aa1	HSP90AA1	-0.42	4.45×10-03
Cdc42bpb	CDC42BPB	-0.64	6.56×10-05
Mta1	MTA1	-0.64	1.29×10-03
Rala	RALA	0.44	5.90×10-03
Slc22a23	SLC22A23	-0.68	1.45×10-04
Tpmt	TPMT	0.53	2.12×10-03
Cetn3	CETN3	1.08	1.94×10-07
Dhfr	DHFR	0.61	1.12×10-03
Fhit	FHIT	1.08	4.97×10-05
Dlg5	DLG5	-0.82	5.84×10-03
Zmiz1	ZMIZ1	-0.77	5.00×10-03
Prkcd	PRKCD	-0.59	7.27×10-03
Gch1	GCH1	0.57	2.50×10-05
Pnp	PNP	0.47	5.01×10-03
Mmp14	MMP14	-0.44	4.15×10-03
Clu	CLU	-0.48	1.32×10-04
Vwa8	VWA8	-0.69	7.99×10-07
Lifr	LIFR	-0.49	2.77×10-03
Ago2	AGO2	-0.87	3.09×10-05

Plec	PLEC	-0.84	8.40×10-04
Rac2	RAC2	-0.63	6.67×10-03
Lgals1	LGALS1	-0.78	2.80×10-05
Ep300	EP300	-0.62	1.44×10-03
Krt8	KRT8	0.47	4.62×10-04
Ube2l3	UBE2L3	-0.45	3.29×10-03
Eif4g1	EIF4G1	-0.44	1.95×10-03
St6gal1	ST6GAL1	-0.70	9.33×10-07
Cpn2	CPN2	-0.55	1.47×10-04
Tfrc	TFRC	0.67	1.28×10-03
Sod1	SOD1	0.48	3.77×10-03
Arid1b	ARID1B	-0.81	2.04×10-05
Plg	PLG	-0.52	8.85×10-05
Igf2r	IGF2R	-0.84	8.87×10-08
Tapbp	TAPBP	-0.59	9.53×10-05
C4b	C4B	-0.94	2.48×10-07
Prrc2a	PRRC2A	-0.80	1.20×10-04
H2-Q1	HLA-A	1.24	2.66×10-17
Vars2	VARS2	-0.56	5.93×10-03
C3	C3	-0.61	1.61×10-05
Zfp36l2	ZFP36L2	-0.55	6.40×10-03
Ttc7	TTC7A	-0.48	4.40×10-03
Cdh2	CDH2	-0.77	1.88×10-05
Nfatc1	NFATC1	-0.59	4.78×10-03
Lrp5	LRP5	-1.33	1.58×10-07
Rela	RELA	-0.54	1.86×10-03
Syvn1	SYVN1	-0.65	1.30×10-03
Bad	BAD	0.60	1.91×10-03
Dntt	DNTT	0.83	7.50×10-05
Hps1	HPS1	-0.63	5.03×10-03
Abcc2	ABCC2	-0.48	8.13×10-03
Casp7	CASP7	0.46	1.70×10-03
Hprt	HPRT1	0.46	3.13×10-03
Cetn2	CETN2	0.61	2.51×10-03
Cldn2	CLDN2	-0.94	1.32×10-06
Sat1	SAT1	0.63	2.41×10-05
Prdx4	PRDX4	0.46	2.82×10-03
Tmsb4x	TMSB4X	0.45	8.13×10-03

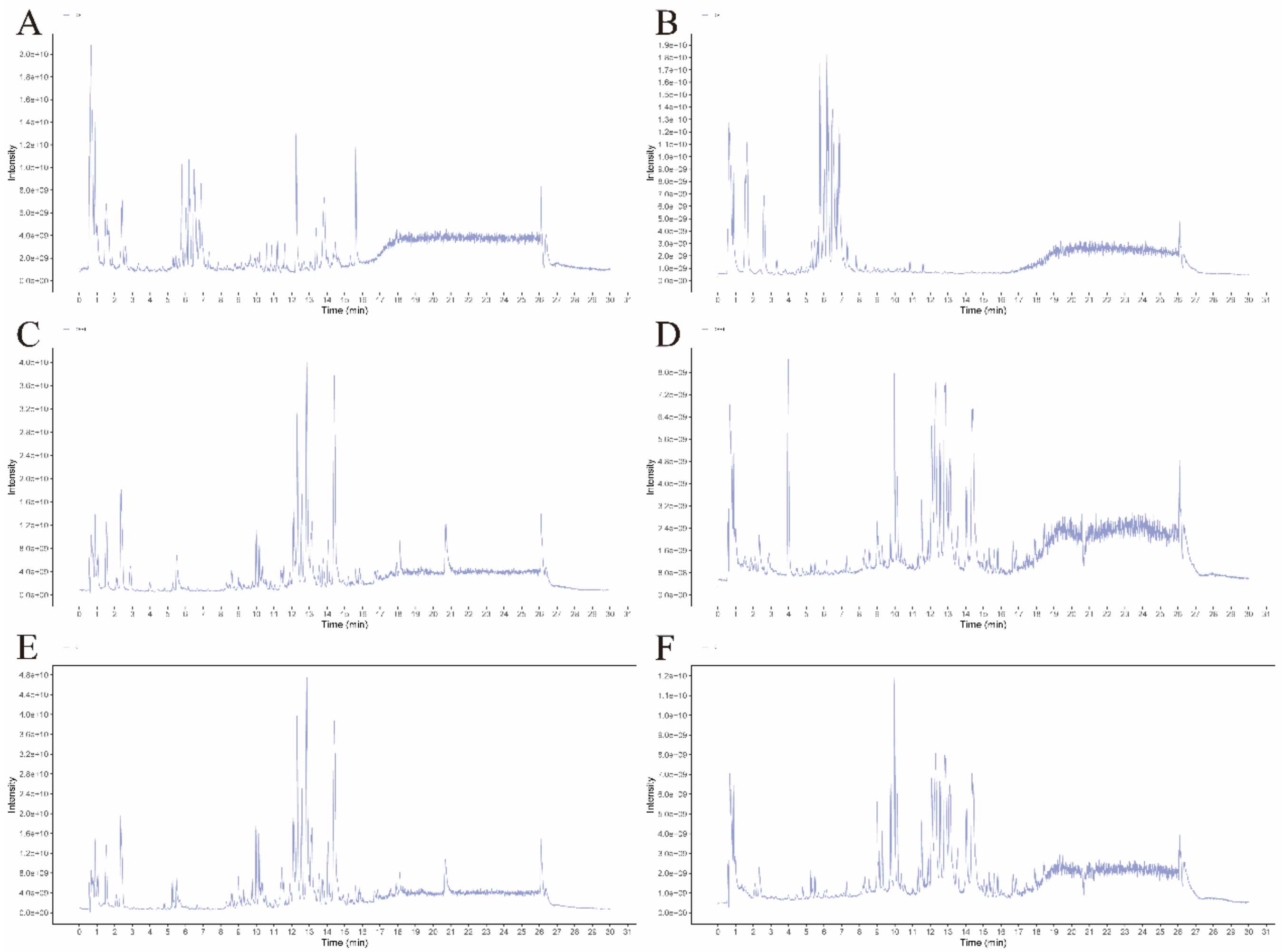

Figure S1. Total ion chromatograms were analyzed by UPLC-MS.

(A) Danshen extract positive ion map (B) Danshen extract negative ion map

(C) Danshen-containing serum positive map (D) Danshen-containing serum negative ion map

(E) Blank serum positive ion map (F) Blank serum negative ion map
